# A Comparison of Methods to Measure the Magnetic Moment of Magnetotactic Bacteria through Analysis of Their Trajectories in External Magnetic Fields

**DOI:** 10.1371/journal.pone.0082064

**Published:** 2013-12-12

**Authors:** Rohan Nadkarni, Solomon Barkley, Cécile Fradin

**Affiliations:** 1 Department of Biochemistry and Biomedical Sciences, McMaster University, Hamilton, Ontario, Canada; 2 Department of Physics and Astronomy, McMaster University, Hamilton, Ontario, Canada; Dalhousie University, Canada

## Abstract

Magnetotactic bacteria possess organelles called magnetosomes that confer a magnetic moment on the cells, resulting in their partial alignment with external magnetic fields. Here we show that analysis of the trajectories of cells exposed to an external magnetic field can be used to measure the average magnetic dipole moment of a cell population in at least five different ways. We apply this analysis to movies of *Magnetospirillum magneticum* AMB-1 cells, and compare the values of the magnetic moment obtained in this way to that obtained by direct measurements of magnetosome dimension from electron micrographs. We find that methods relying on the viscous relaxation of the cell orientation give results comparable to that obtained by magnetosome measurements, whereas methods relying on statistical mechanics assumptions give systematically lower values of the magnetic moment. Since the observed distribution of magnetic moments in the population is not sufficient to explain this discrepancy, our results suggest that non-thermal random noise is present in the system, implying that a magnetotactic bacterial population should not be considered as similar to a paramagnetic material.

## Introduction

Magnetotactic bacteria are a diverse, paraphyletic group of prokaryotes that demonstrate magnetotaxis, or movement along magnetic fields 1,2. Since their discovery in 1975, they have been identified in freshwater and marine ecosystems worldwide [3]–[6]. Magnetotaxis is accomplished through the synthesis and accumulation of magnetosomes, which are membrane-bound organelles containing crystals of iron oxides or iron sulfides [7]. The magnetosome chain is fixed within the cell and so interactions of its magnetic dipole moment, *μ*, with an external magnetic field, *B*, affect the entire bacterium [3], [8]. A uniform magnetic field induces a torque that tends to align cells with local magnetic field lines. Thus, magnetotactic bacteria passively orient with magnetic field lines and actively travel along them by rotating their flagella. This is believed to facilitate the efficient movement of bacteria towards favourable environmental conditions that are partially correlated with the direction of the geomagnetic field [9], [10]. Specifically, magnetotaxis appears to improve detection of vertical gradients of dissolved oxygen concentration [11], [12], which is consequential as the majority of these bacteria are anaerobes or microaerobes [10], [13].

Some of the most well studied magnetotactic bacteria are members of the *Magnetospirillum* genus, which are freshwater biflagellate spirilla that incorporate magnetite (Fe_3_O_4_) into a single linear chain of magnetosomes [14]. Axial magnetotaxis has been reported for magnetospirilla, where cells are able to swim in both directions along the magnetic field lines with frequent reversals in the direction of motion not requiring magnetic reorientation [9], [12]. Within this genus, *Magnetospirillum magneticum* strain AMB-1 is frequently used as a model for magnetotactic bacteria (e.g. [8], [11], [15]–[19]). This strain was isolated in Tokyo and first described in 1991 [20]. Its genome was sequenced in 2005 [15]. Three different measurements of the average magnetic moment of a population of AMB-1 cells have been reported so far. Its value has been estimated as 1×10^−16 ^A⋅m^2^ based on iron uptake [11], 0.7×10^−16 ^A⋅m^2^ using vibrating sample magnetometry [19] and 0.5×10^−16 ^A⋅m^2^ using optical magnetic imaging [18].

There are several other ways to measure the magnetic moment of magnetotactic cells. One of the most common methods is to sum the magnetic moments of all the magnetic crystals found within a cell, as calculated using the known properties of magnetite and the crystals’ dimensions measured in transmission electron micrographs [2], [4], [21]. This method is often used to confirm the validity of other less direct measurement techniques (eg. [22]–[25]). Another well-documented method, the “U-turn” method, consists in calculating the magnetic moment of a cell from its response time after a sudden magnetic field reversal [4], [22]. Other single cell measurements of cellular magnetic moments come from more involved studies employing direct measurement techniques such as superconducting quantum interference device (SQUID) magnetometry [25] and electron holography [26]. Another group of methods, referred to here as “statistical methods”, infer the magnetic moment from observed distributions of cellular orientation for a population of cells. This type of analysis has been performed previously on distributions measured from cells’ mean direction of travel [23] and from the scattering and birefringence of light [24], [27].

In this study, we describe five novel methods to measure magnetic dipole moments based on the analysis of bacterial trajectories subjected to external magnetic fields. The advent of affordable high frame rate cameras and the development of tracking algorithms have rendered such methods easy to implement. Three of these methods are statistical, inferring the magnetic moment from orientation distributions, and the other two are based on viscous relaxation dynamics in response to orientation perturbation. Some represent improvements on methods reported previously (for example the “U-turn” method), while others are new. We show here that these methods can be implemented using very low cost lens and camera. All of our measurements were performed on the same population of *M.* *magneticum* AMB-1 bacteria, allowing a direct comparison between the values of the magnetic moments obtained using trajectory-based methods and that obtained using the established technique of magnetosome dimension estimate from electron microscopy images. We find that statistical methods return values of the magnetic moment that are systematically lower than other methods, suggesting that the cell’s orientation might be influenced by non-thermal stochastic forces.

## Theory

### Distribution of Cell Orientations and the Paramagnetic Model

**Figure 1:**
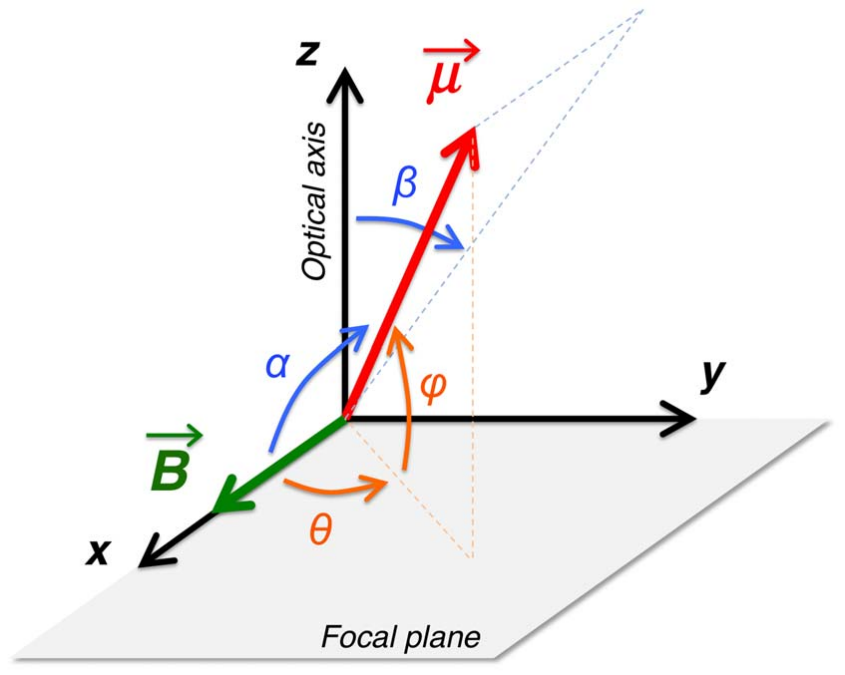
Cell orientation with respect to the magnetic field and the optical axis. Two different spherical coordinates systems were used to describe cell orientations (assimilated to the direction of their magnetic moment). In the first coordinate system (blue), the zenith direction is the direction of the magnetic field (x-axis), *α* is the polar angle (and the actual angle between 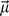 and 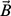) and *β* is the azimuth angle. In the second coordinate system (orange) the zenith direction is set along the optical axis (z-axis), φ is the complementary angle to the polar angle (and the inclination of the cell out of the focal plane) and θ is the azimuth angle (and the apparent angle between 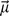 and 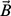 when trajectories are projected in the focal plane).

Magnetotactic bacteria have been described as “self-propelled compass needles,” meaning that their propulsion is distinct and separate from their orientation [10], [28]. The bacteria are usually considered to respond to a magnetic field in a way similar to that of atoms in a paramagnetic material, where the orientation of each magnetic dipole is influenced only by its interaction with the applied external magnetic field and by random thermal fluctuations [21], [23], [29]. This paramagnetic model of magnetotactic bacteria predicts a distribution of orientations that follows Boltzmann statistics and depends on the angle α between the magnetic moment of the cell and magnetic field through:
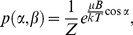
(1)where *Z* is the partition function and the angle *β* is defined in Fig. 1.

The degree of orientation with the magnetic field is usually evaluated by considering the average cosine of the orientation angle, <cos *α*>, which for magnetic dipoles free to move in three dimensions follows the Langevin function [28]:
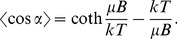
(2)


This quantity is accessible experimentally, as it relates the velocity component in the direction of the magnetic field to the total speed, and Eq. 2 has been used previously to estimate the magnetic moment of individual magnetotactic cells [23]. However, for a population where individual cells move in either direction along magnetic field lines and occasionally reverse direction, <cos *α*> = 0. In order to estimate the average magnetic moment of an axial magnetotactic cell population we considered instead the variance in sin *α,* which provides a measure of the dispersion of cell orientations relative to constant magnetic field lines, and should decrease with magnetic field strength:
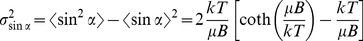
(3)


Having said that, it is not the actual orientation of the cells with respect to the magnetic field (*α*) that is observed when recording the motion of cells in the focal plane of a microscope, but instead the projection of this orientation along the optical axis (*θ*, as depicted in Fig. 1). The apparent angular distribution for cells with three-dimensional trajectories but for which only two-dimensional projections of these trajectories should be, according to the paramagnetic model:
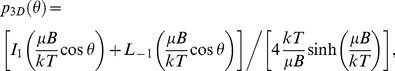
(4)where *I_n_* and *L_n_* are the modified Bessel and modified Struve functions of the first kind of order *n*. This distribution is obtained by recognizing that 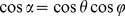 (the angle *φ* is the inclination of the cell out of the focal plane, as represented in Fig. 1) and by integrating Eq. 1 over all possible values of *φ*. Similarly, the variance of sin*θ* for cells with three-dimensional trajectories can be shown to be:



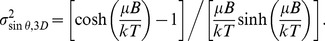
(5)Eqs. 4 and 5 are obtained by assuming that a cell can adopt any orientation in three dimensions (although some are favored because of the interaction of their magnetic moment with the magnetic field). The cells observed in our experiments, however, seemed to have a motion that was largely restricted to the focal plane, as discussed in the results section. For two-dimensional motions (as opposed to projected three-dimensional motions) the paramagnetic model predicts a distribution of cell orientations:
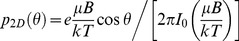
(6)


A two-dimensional version of equation 3 can also be derived to assess the alignment of magnetic moments with the magnetic field in the case of true two-dimensional trajectories, by integrating p(*θ)*sin*θ* and p(*θ)*sin^2^ *θ* over all possible orientations in a plane:
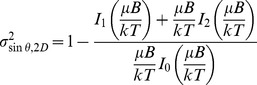
(7)where I_n_ is the modified Bessel functions of the first kind of order n.

Additionally, we considered the possibility that the bacteria exhibit a normal distribution of magnetic moments, 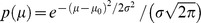, with mode μ_0_ and standard deviation *σ*. The distribution of orientations is then no longer given by equation 6, and instead becomes (for two-dimensional trajectories):
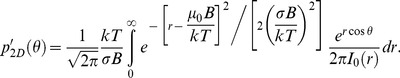
(8)


### Cell Rotation and the Bean’s Model

The behaviour of a magnetotactic cell following a sudden reversal of the magnetic field has been described previously. The equations describing this process have been attributed to Bean (e.g. [28], [30]), but were most clearly laid out by Esquivel and Lins de Barros [4]. Since bacterial swimming takes place at a very low Reynolds number, inertial terms can be omitted when writing the torque equation for a single cell. On average, random forces will create a zero net torque, so the rotational drag must exactly balance the torque exerted by the magnetic field on the dipole and the torque equation reduces to:
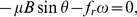
(9)where 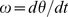 is the cell angular velocity and *f_r_* is the rotational drag coefficient, which depends on the size and shape of the cell. Eq. 9 can be integrated to obtain the orientation of the cell as a function of time:
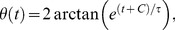
(10)where 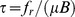 is the characteristic relaxation time and C is a constant of integration defined by the angle at time t = 0. Esquivel and Lins de Barros proceed by assuming that initial orientation is governed by Boltzmann statistics in order to calculate an approximate value for C, and obtain expressions for the reversal time and the reversal diameter, two easily accessible quantities [4]. This procedure has often been used to measure the magnetic moment of magnetotactic organisms [4], [22], [31], [32].

To avoid any dependence on the paramagnetic model, we have instead left the integration constant *C* as a free parameter, and we have analyzed full trajectories instead of just considering reversal time and diameter. For a cell undergoing a single field reversal at time *t* = 0, we considered the quantity:
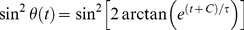
(11)which does not depend on whether the cell moves in the direction of the magnetic field or against the magnetic field, and whether it turns left or right upon magnetic field reversal. Fitting this quantity while leaving *C* as a free parameter allows retrieval of the magnetic moment of the cell. In practice, we used a periodically reversing magnetic field (with period *T*, so that the field was reversed every *T*/2), so that we should expect:



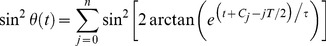
(12)The fact that there is a different integration constant, *C_j_*, for each field reversal reflects the fact that due to thermal noise the motion of the bacteria is not truly periodic even though the magnetic field variations are.

A fit to Eq. 11 or 12 returns the relaxation time 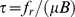, thus the value of the rotational drag coefficient *f_r_* is needed in order to measure *μ*. For a sphere of radius *R* rotating in a medium of viscosity *η,*

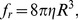
(13)which has been used as an estimate for the rotational drag coefficient of magnetotactic cells previously [4]. Another method involves dividing each cell into a chain of spheres to better represent the shape of magnetospirilla [22], [30]. However, this approximation was developed for cells with a length to diameter ratio of approximately 7 [22], whereas the ratio of those studied here is approximately 3. Instead, cells were modeled here as cylinders rotating perpendicular to their axis (as done previously for example by Chemla et al. [33]), for which a good approximation of the rotational drag coefficient is given by:
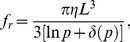
(14)where *p* is the length to diameter ratio of the cylinder, *L* is its length, and δ(*p*) is a correction for end effects which depends on the cylinder aspect ratio [34].

### Orientation Correlation and the Worm-like Chain Model

The orientation correlation of a two-dimensional trajectory is defined as 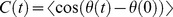, where *θ*(t) defines the direction of the tangent to the trajectory at time *t*. Cellular trajectories are sometimes compared to the path of a flexible polymer, and in particular to that of polymers described by the worm-like chain (WLC) model [35]. By definition, the orientation correlation function for WLC trajectories decays exponentially according to 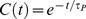. The persistence time of the trajectory, *τ_P_*, depends on the torques applied on the cell. In the absence of a magnetic torque, the balance between thermal and viscous forces leads to 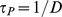, where 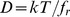 is the rotational coefficient of the cell. For a magnetotactic cell in the presence of a magnetic field, however, we expect the persistence time to decrease and to depend also on the characteristic relaxation time 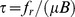.

In the WLC model there is no correlation between the initial and long-time orientations of the path, in other words 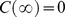. The situation is different for magnetotactic bacteria exposed to an external magnetic field, since their orientations remain correlated at all time through the magnetic field. We thus expect an orientation correlation function of the form:
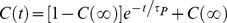
(15)


If *θ* is the angle between the tangent to the trajectory observed in the focal plane and the magnetic field, it is also the angle between the projected magnetic moment of the cell and the magnetic field, and it follows that the long-time value of the orientation correlation function can be expressed as 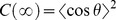. This value can be made more explicit if one assumes that the orientation of bacteria is governed by Boltzmann statistics according to the paramagnetic model. If the cells are free to move in three dimensions, then 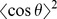 can be calculated by integrating *p*(*θ,φ)*cos^2^ *θ* over both angles *θ* and *φ*, giving:
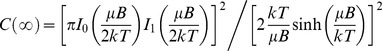
(16)


However, if the cells are only able to take orientations in two dimensions, only one integration (over *θ*) is necessary in order to calculate 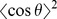, and the paramagnetic model predicts:
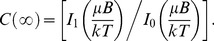
(17)


## Methods

### Cell Culture

The bacterial strain *Magnetospirillum magneticum* AMB-1 (ATCC 700264) was obtained from the American Type Culture Collection. For cell culture revised Magnetic Spirillum Growth Medium (MSGM) was prepared in-house according to the recipe provided by ATCC (ATCC Medium 1653) using Wolfe’s Vitamin Solution (ATCC MD-VS) and Wolfe’s Mineral Solution (ATCC MD-TMS), both purchased from ATCC. Immediately after preparation, the medium was sterilized by passage through a 0.2 µm membrane (Acrodisc 25 mm syringe filter) and stored at 4°C until use. Cells were grown at 25°C in airtight 15 ml Falcon tubes entirely filled with MSGM in order to achieve microaerobic conditions. Every 7 to 10 days, cells were centrifuged and resuspended in fresh MSGM medium after the supernatant was discarded. Then 1.5 ml of the resuspended solution was transferred to a new Falcon tube and supplemented with fresh MSGM so that the tube was entirely filled. When cells were needed for observation, a small volume of a 7-day old culture was removed from the layer placed just above the cell sediment that always formed at the bottom of the Falcon tube, which was found to contain the highest concentration of live cells, and diluted in MSGM as needed to obtain the desired cell concentration.

### Transmission Electron Microscopy

Transmission Electron Microscopy (TEM) images of individual cells were obtained by placing ∼5 µl of suspended bacteria on Formvar-coated TEM grids, followed by negative-staining with 1% aqueous uranyl acetate. Grids were viewed in a JEOL JEM 1200 EX TEMSCAN transmission electron microscope (JEOL, Peabody, MA, USA) operated at an accelerating voltage of 80 kV. A 4-megapixel digital camera (Advanced Microscopy Techniques Corp, Dancers, MA, USA) was used to capture images. The total magnetite volume present in a cell was estimated as follows. First, the lengths of four magnetite crystals were measured along the axis of the magnetosome chain from the images using ImageJ [36]. Crystals were selected to include those at either end of the chain as well as two adjacent crystals near the middle of the chain. The volume of each of the measured crystals was then calculated assuming a spherical shape. The mean crystal volume was calculated for each cell and the total magnetite volume was estimated by multiplying this mean by the total number of crystals in the magnetosome chain.

### Recording of Cell Trajectories in Presence of an External Magnetic Field

For movie recording, ∼15 µl bacteria suspended in MSGM medium were placed in a thin sample chamber assembled from a microscope slide and coverslip and sealed with wax. The bacteria were then immediately imaged using a Nikon Eclipse TS100 microscope and a 40× dry objective with NA 0.55, which allowed sufficient optical resolution while ensuring a large enough field of view to record long cell trajectories. Phase contrast movies were recorded using a Moticam 1000 camera and the software Motic Image Plus 2.0 (Motic, BC, Canada). Frame dimensions were 640×512 pixels (pixel size 8 µm in the image plane, corresponding to a pixel size of 0.20 µm in the sample plane) and mean frame rate varied between 20–22 fps for each movie. A horizontal magnetic field was generated using two Helmholtz coils (3.6 cm coil radius, 4 cm coil spacing, 300 wire loops per coil, copper wire with 0.06 mm^2^ cross-section) and reinforced by two cylindrical iron cores (60 mm length, 10 mm diameter). Current in the coils was provided by a 6614C DC power supply (Agilent, Mississauga, ON, Canada). A 1-HS DC Gaussmeter (AlphaLab, Utah, USA) was used to calibrate the magnetic field strength, which could be varied between ∼0 and 5.6 mT in the center of the sample.

### Tracking of Individual Cells

Bacteria were tracked manually using the MTrackJ plugin for ImageJ [37]. To avoid user bias when choosing cells for tracking, a frame was selected near the middle of each movie and all cells present within a central rectangular region (80 µm×50 µm) were tracked along their entire trajectories. This procedure was repeated using a different frame at a later time in the movie until a sufficient number of trajectories were acquired (on average 33 cells were tracked at each magnetic field value). For some selected movies, the length of each tracked cell was also estimated, by measuring the length five times in different frames spaced evenly along the entire trajectory of the cell. The resolution of the instrument, limited by diffraction, was only ∼0.5 µm due to the relatively low numerical aperture of the objective lens that was used. However, because the image was oversampled and because the cylindrical shape of the bacteria is symmetric, the position of the center of mass of the cells and their length could be determined with a precision of 1 pxl = 0.2 µm. Subpixel resolution for phase contrast images of bacteria can be achieved using tracking algorithms, as demonstrated for example in Ref. [38]. However it was not attempted here since manual tracking gave sufficient precision for our application.

### Trajectory Analysis

Analysis of the trajectory data collected using MTrackJ was done using Mathematica (Wolfram Research, IL, USA). From the position of the cells and the known direction of the magnetic field, the orientation of their trajectories between each two consecutive frames (i.e the angle *θ*) was calculated at each time point for each tracked cell. The data was then filtered to eliminate spurious or unwanted trajectories or steps in trajectories. Specifically, a cell that moved less than 0.5 µm between consecutive movie frames was considered stationary at that time and the orientation of the trajectory measured between these two frames was rejected as unreliable. Also, some of the cells that were tracked in the constant magnetic field movies did not demonstrate magnetotaxis and produced irregular trajectories, which often included frequent loops or frequent random changes of direction. These can be distinguished from the regular, linear trajectories by examining the standard deviation of the sine of the trajectory angle, which is much higher for irregular tracks. All tracks with a standard deviation of the sine of trajectory angles that was greater than 0.55 (between 0 and 18% of trajectories in each video with an imposed external magnetic field) were excluded from subsequent analyses.

### Rotational Bias

From trajectories recorded for a certain constant magnetic field, the value of the average angular velocity of the cells as a function of their orientation was calculated as follows. For each cell at each time point the orientation with respect to the direction of the magnetic field (without any distinction between cells traveling parallel or antiparallel to the magnetic field, so that −90° ≤ θ ≤ 90°) was rounded to the nearest 10°. The associated angular velocity, *ω,* was estimated as the change in orientation between the current frame and the next frame multiplied by the average frame rate of the movie.

### Orientation Distribution

To calculate the variance of the quantity sin*θ*, we first calculated the average value of sin*θ* for each cell along its complete trajectory, <sin*θ*>, and then calculated the variance of <sin*θ*>, noted *σ* ^2^_<sin*θ>*_, for the population of cells tracked at each particular value of the magnetic field. This helped reduce measurement noise and avoided placing excessive weight on cells with longer trajectories.

### Orientation Correlation

Before an orientation correlation function could be calculated from trajectories, it was necessary to detect and account for spontaneous reversals in the direction of motion which occurs for axial magnetotactic bacteria such as *M. magneticum*. In the context of orientation correlation, reversals represent an immediate negative correlation that is not due to alignment with the magnetic field and follows no predictable temporal pattern. Reversal events were identified as points along a trajectory for which the mean angle of the five preceding points and the mean angle of the five subsequent points were within 30° of complete opposition. For each magnetic field value, all uninterrupted portions of trajectories between reversals, referred to as segments, were then used to calculate the orientation correlation function. To reduce the dependence of the data on the behavior of individual cells, orientation correlation functions were calculated only for time-points with information available from at least 6 cells.

## Results

### Estimation of Magnetic Moment from Magnetosome Dimension

**Figure 2:**
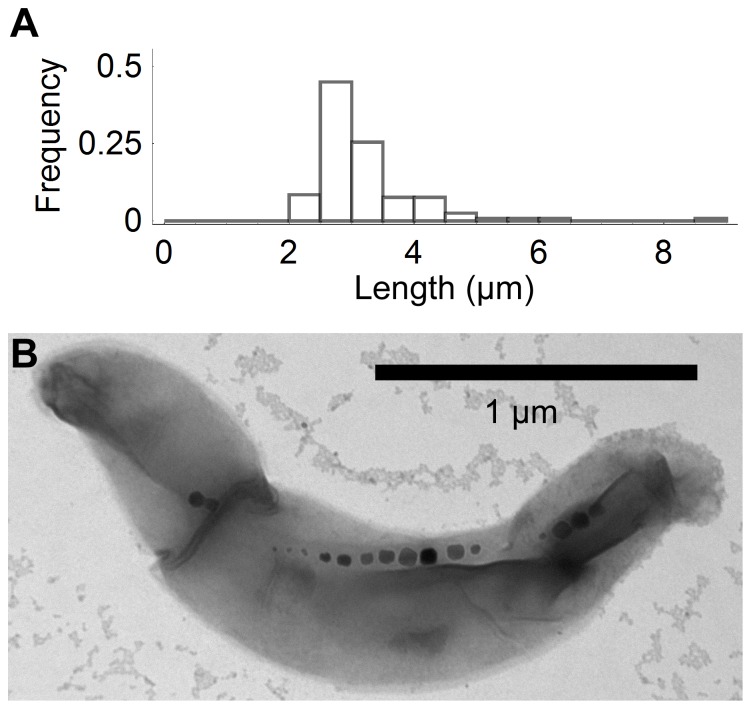
Dimensions of *M. magneticum* cells and magnetosomes. A) Distribution of cell lengths as measured from 118 cells observed under the light microscope. The reported length of each cell is an average of five measurements done in different movie frames. B) Transmission electron micrograph of a typical cell. Magnetosome dimension estimate from the image returns a value of the magnetic moment *μ* = 4.3×10^−16^ A⋅m^2^ for this cell.

*M. magneticum* cells are spiral shaped and about 3 µm in length. The size distribution of the cells in our culture, as measured from phase contrast microscopy images, is shown in Fig. 2A. The distribution is right-skewed, with a mean length of 3.21 µm. The mean standard error when measuring the size of each cell was 0.2 µm (for n = 5 measurements per cell), in agreement with the expected ∼1px = 0.2 µm precision on bacterial length and position, and significantly smaller than the standard deviation obtained for the lengths of the population, which was 0.9 µm (for n = 118 cells).

Although they cannot be resolved with light microscopy, magnetosomes are clearly visible using transmission electron microscopy (Fig. 2B). We estimated the total volume of the magnetosomes as seen in transmission electron micrographs for 22 different cells, as explained in the methods section. The measured magnetite crystals had a mean diameter of 42 nm and a mean volume of 5.1×10^4^ nm^3^. Cells had an average of 17 magnetosomes, with a standard deviation of 6 magnetosomes. Using the known magnetic moment per unit volume of magnetite, 4.8×10^−22^ A⋅m^2^/nm^3^ [39], the magnetic moment of the cells could be estimated from the total magnetosome volume. The mean magnetic moment was 4.2×10^−16^ A⋅m^2^ with a standard error of 0.5×10^−16^ A⋅m^2^. For this population, we found no obvious correlation between the value of the magnetic moment and the length of the cells.

### Estimation of Magnetic Moment from Cell Trajectories under Magnetic Field Reversal

**Figure 3:**
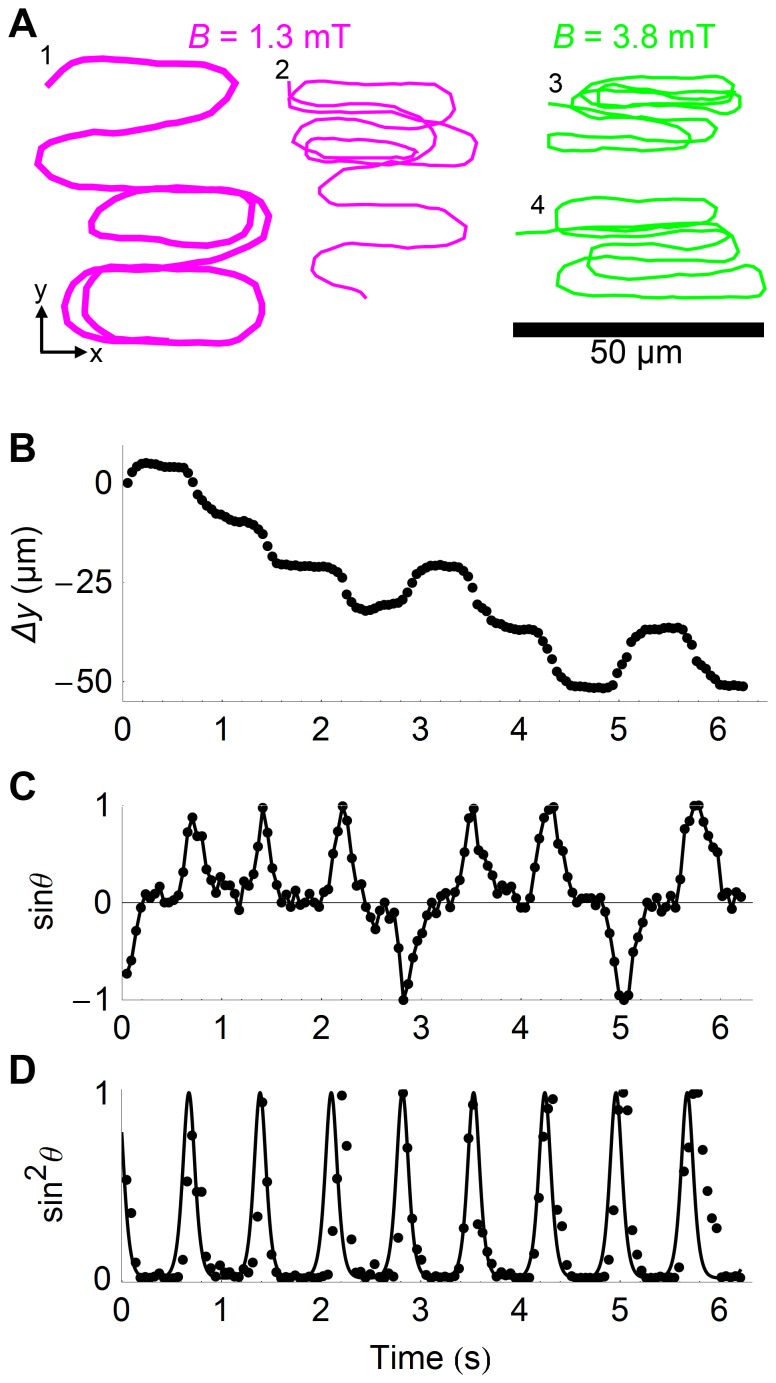
Cell trajectories in periodically reversing magnetic fields. A) Trajectories of four cells moving in response to a periodically reversing magnetic field of frequency 700 mHz and amplitude indicated by trace colour. Subsequent analysis of trajectory 1 is presented in panels B, C & D. B) Position of the cell in the direction perpendicular to that of the magnetic field. C) Sine of the angle between the direction of movement of the cell and the magnetic field. D) Square of the sine of the angle plotted in panel C. Fit to Eq. 12 yields a magnetic moment *μ* = 3.2×10^−16^ A⋅m^2^ for this cell, as explained in the text.

One of the most popular methods to estimate magnetic moment from cell trajectories is the so-called “U-turn method”, where cells are submitted to a series of magnetic field reversals [4], [22], [32]. The cell responds to each reversal by completing a U-turn during which it rotates 180 degrees, each time choosing a random direction (left or right), as shown in Fig. 3A. Both the diameter of the U-turns, which can be visualized by looking at the displacement of the cell perpendicular to the magnetic field (Fig. 3B), and their duration, which can be visualized by looking at the sine of the cell orientation (Fig. 3C), depend on the quantity *µB* and can be used in principle to measure the magnetic moment. However, here only projections of the motion in the focal plane are observed, therefore the observed diameter might be smaller than the actual U-turn diameter [13], [28], [30]. Therefore it is more reliable to obtain *μ* from the cell’s response time.

Extracting *μ* from the measured value of the response time to a field reversal, as is usually done, requires assumptions about the average orientation of the cells under a constant magnetic field (see theory section for details). Instead we considered the full cell trajectory by fitting the quantity *sin^2^θ* using Eq. 12 (Fig. 3D). This parameter is approximately zero when the cells are moving parallel to the magnetic field between field reversals, but positive peaks occur whenever the magnetic field reverses. We found that the motion was in general well enough approximated as being periodical, although small variations can be observed, so we fit the data using a single value for the constants *C_i_*. For each cell, the fit returned the characteristic relaxation time *τ* = *f_r_/(μB)*, from which the value of the magnetic moment was extracted using the known magnetic field value and an estimate of the rotational drag coefficient. We assumed cells were cylinders (Eq. 14) of length 3.21 µm and aspect ratio *p = *3.3 (end effects correction *δ*(3.3) = −0.392 [34]) in a medium with viscosity equal to that of water (*η = *1.002 mPa⋅s [40]), yielding *f_r_* = 4.26×10^−20^ kg⋅m^2^/s. We analyzed the trajectories of 31 different cells, recorded in different field conditions (magnitude *B* = 1.3, 2.0 or 3.8 mT and period *T = *1.4 or 2 s). The mean magnetic moment obtained from this analysis was 4.3×10^−16^ A⋅m^2^ with a standard error of 0.4 A⋅m^2^, with no obvious dependence on either magnetic field strength or period.

### Estimation of Magnetic Moment from Cell Trajectories in Constant Magnetic Fields

#### General considerations

**Figure 4:**
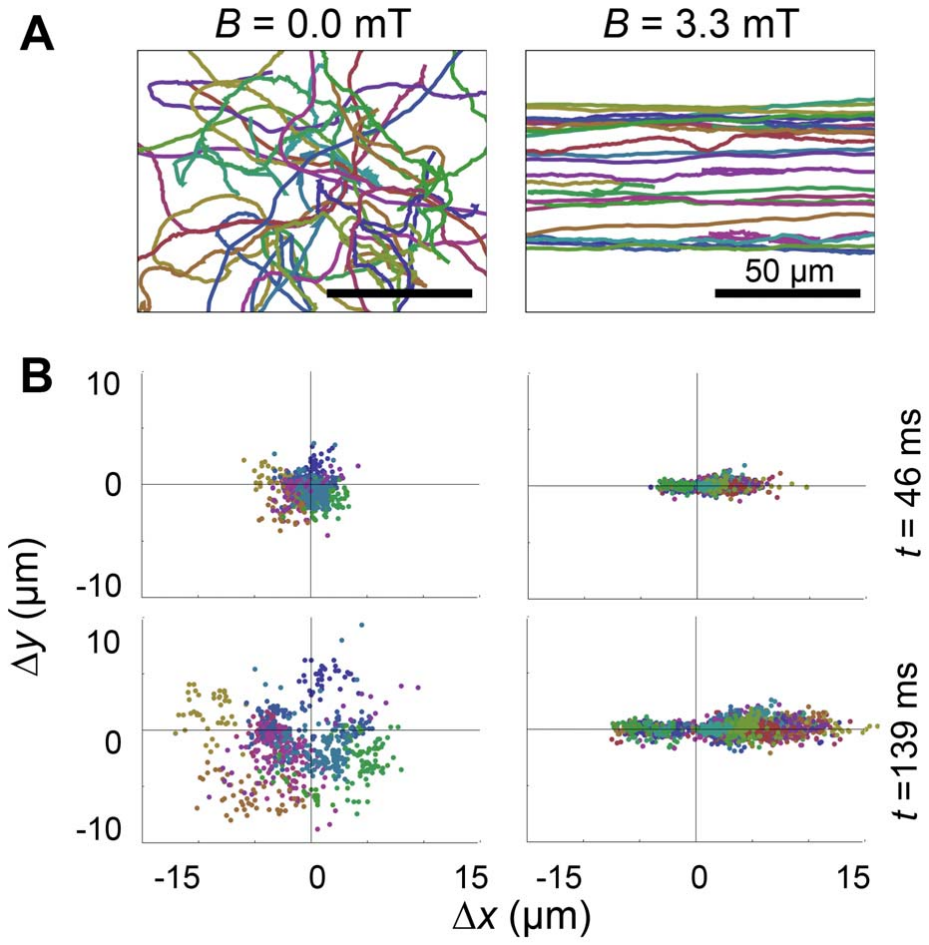
Cell trajectories in constant magnetic fields. A) Trajectories of 25 cells in a zero (left) and non-zero (right, *B = *3.3 mT) magnetic field. B) Displacement of the cells after *t* = 46 ms (upper panels) and *t* = 139 ms (lower panels). Only regular trajectories used in subsequent analyses are presented here, with data for *B* = 0 mT (left panels, 10 cells) and *B = *3.3 mT (right panels, 51 cells). Each different colour represents a different cell, with the displacement of each cell measured over multiple time intervals.

Movies of cells placed under a constant magnetic field with a magnitude chosen between *B = *0 and 6 mT were recorded. About 30 cells were tracked in each movie, yielding sets of trajectories such as the ones shown in Fig. 4A. Even in the absence of an external magnetic field, the cell trajectories appeared to be restricted to a thin layer above the glass coverslip surface, as evidenced by the fact that most cells were found within ∼20 µm of either the lower or upper glass surface. Further evidence that the trajectories we observed were in fact quasi two-dimensional was that in the large majority (∼80%) of cases individual cells could be tracked without interruption until they left the field of view, and that the apparent length of the cells in the movies (i.e. their projected length in the focal plane) was mostly within 15% of their maximum length, suggesting that the inclination of the cells out of the focal plane was never more than 30%. A preference for bacteria to stay in the vicinity of glass surfaces as been documented already for *E. coli*, and attributed to either an attractive interaction potential between the cells and the surface or hydrodynamic interactions [41], [42].

The alignment of the cell trajectories with the magnetic field is obvious at high magnetic field. The corresponding distribution of displacements obtained after 1 frame (t = 46 ms) or 3 frames (t = 139 ms) is shown in Fig. 4B. At low magnetic field values a “doughnut” shape distribution is observed after about 100 ms (Fig. 4B, left panels). This indicates that the persistence length of the cell trajectories was larger than 100 ms, and at least comparable to the scale of our measurements, where each cell was typically detected for a few seconds. Therefore single cells may not sample all possible orientations during a single passage in the field of view, so that in the conditions of our experiments statistical measurements required examining a population of cells rather than a single cell along its trajectory. At high magnetic field values a “bow tie” shape distribution is observed, illustrating the fact that the cells may travel in a direction that is either parallel or anti-parallel to the direction of the magnetic field, as expected for magnetospirilla. Sudden trajectory reversals are observed both at low and high fields.

Before the analysis of the cell trajectories was performed, the data were filtered in several ways to remove irregular trajectories and data points corresponding to uncertain cell orientation, as explained in the methods section. For the remaining points of the remaining trajectories, the speed and direction of the trajectory was calculated for each pair of consecutive points. The cells exhibited a speed with a mean of 44 µm/s and standard deviation 18 µm/s. There was no obvious correlation between speed and external magnetic field value, or speed and cell length, in agreement with what has been previously reported [17].

#### Rotational bias

**Figure 5:**
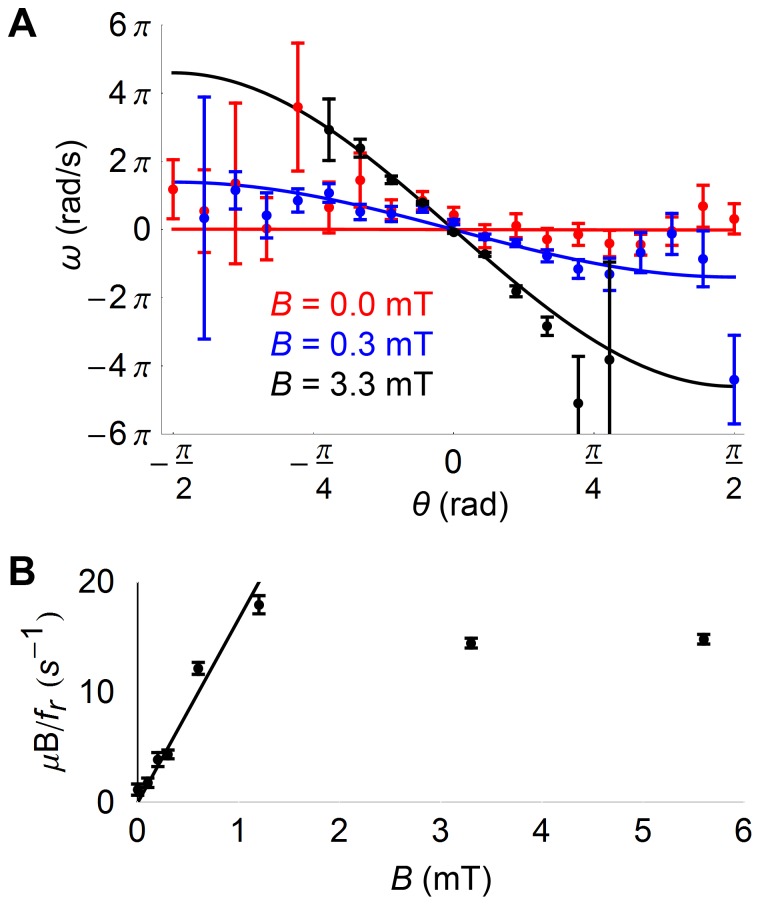
Rotational bias of cells placed in constant magnetic fields. A) Average angular velocity of cells as a function of their initial orientation, plotted for magnetic fields with different magnitudes (mean ± standard error). Fit was performed according to the rotational bias model (Eq. 9). B) Ratio of magnetic torque to rotational drag coefficient for cells placed in magnetic fields of various magnitudes. Plotted values are obtained from fit of data such as those shown in panel A. Linear fit to the data measured at magnetic fields *B*<2 mT yields a magnetic moment *μ* = 5.7±0.2×10^−16^ A⋅m^2^, as explained in the text.

An analysis equivalent to that of the U-turn method can be performed for cells exposed to a constant magnetic field, where instead of the large-scale perturbation provided by the magnetic field reversal one considers the small-scale perturbations generated by thermal noise. Because of thermal noise, the orientation of the cells generally deviates from that of the magnetic field, and one can then examine the “rotational bias”, or the value of the mean instantaneous angular velocity of the cells, <*ω>,* as a function of their orientation, *θ*. The rotational bias should follow Eq. 9 (assuming the cell describe two-dimensional or quasi-two dimensional trajectories in the focal plane). Here the instantaneous angular velocity was calculated from the change in orientation between two consecutive time frames. This is a good approximation as long as the characteristic relaxation time of the bacteria *τ* is large compared to the interval between successive frames, i.e. *B*<2 mT. For B>2 mT, the magnetic torque is large enough to rotate cells to complete alignment with the magnetic field between two frames. As a result, the measured change in angle between frames provides an underestimate of the cell’s instantaneous angular velocity. From the sets of trajectories obtained at each magnetic field value, a mean angular velocity was calculated for each subset of cells with orientations within 10° of each other (Fig. 5A). Each rotational bias curve was fitted directly with Eq. 9, yielding an estimate for the characteristic time *τ* = *f_r_/µB* at each magnetic field value. For *B*<2 mT, the quantity 1/*τ* (plotted in Fig. 5B) increases linearly with magnetic field strength as expected. The slope of the line best fitting the data corresponds to a magnetic moment value of *μ* = (7.1±0.2)×10^−16^ A⋅m^2^.

#### Distribution of cell orientations

The non-interacting magnetic atoms or molecules found in paramagnetic materials have an orientation distribution that follows a Boltzmann distribution (Eq. 1) and an overall orientation, captured by the quantity <cos*α*>, that can be described by a Langevin equation (Eq. 2). Since the cells considered here move both parallel and anti-parallel to the magnetic field, we considered instead the variance of sin*α*, *σ* ^2^_sin*α*_, a quantity that also provides a measure of the alignment with the magnetic field and decreases to zero for a perfectly aligned population of particles (Eq. 3). In addition, the cells tracked in our movies were followed only while in the focal plane, thus it is not the actual angle *α* between 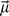 and 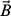 that was detected, but instead the apparent angle *θ* (see Fig. 1). Depending on whether the cells have three-dimensional or two-dimensional trajectories, the paramagnetic model then predicts slightly different orientation distribution for the cells (*p*(*θ*), given by Eq. 4 for 3D trajectories and Eq. 6 for 2D trajectories), and slightly different values of the variance of sin*θ* (*σ* ^2^_sinθ_, given by Eq. 5 for 3D trajectories and Eq. 7 for 2D trajectories).

**Figure 6:**
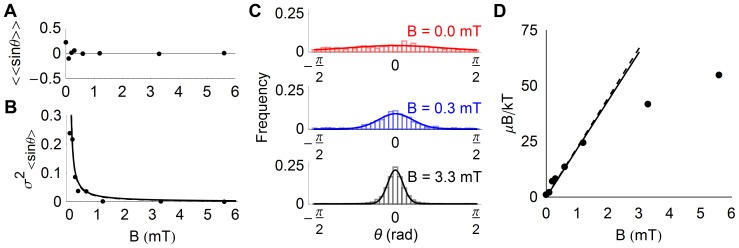
Cell orientation distributions in constant magnetic fields. A) Mean orientation of the cells evaluated as the mean sine of the angle between the observed direction of movement in the focal plane and that of the magnetic field, <<sin*θ*>>. B) Variance of <sin*θ*>. Lines correspond to the expectation of the paramagnetic model: The solid line is a fit assuming 2D trajectories (Eq. 7, yielding *μ* = 1.9×10^−16^ A⋅m^2^), while the dashed line is a fit assuming 3D trajectories (Eq. 5, yielding *μ* = 2.1×10^−16^ A⋅m^2^). C) Distribution of cell orientations for three different values of the magnetic field. Fits are Boltzmann distributions for a monodisperse cell population according to the paramagnetic model for 2D trajectories (Eq. 6). D) Ratio of magnetic energy to thermal energy of the cells obtained from the fit of orientation distributions such as those shown in panel C, assuming either 2D trajectories (black symbols), or 3D trajectories (grey symbols, not visible on the graph as they overlap with the previous ones). Linear fit of the data measured at magnetic fields *B*<2 mT yields a magnetic moment *μ* = 0.90±0.05×10^−16^ A⋅m^2^ assuming 2D trajectories (continuous line) and *μ* = 0.92±0.05×10^−16^ A⋅m^2^ assuming 3D trajectories (dashed line).

We extracted *σ* ^2^_sin*θ*_ from the observed cell trajectories by first calculating the average value of sin*θ* for each cell, and then calculating the mean (Fig. 6A) and variance (Fig. 6B) of the set of values of <sin*θ*> obtained at each particular magnetic field value. As expected, the average value of <sin*θ*> is zero at all field values confirming that there is no bias in the component of motion perpendicular to the field (Fig. 6A). On the other hand, *σ* ^2^_sin*θ*_ decreases with increasing magnetic field in a way that is consistent with the paramagnetic expectation, whether assuming 2D trajectories (Eq. 7, continuous line in Fig. 6B) or 3D trajectories (Eq. 5, dashed line in Fig. 6B). The analysis yields a magnetic moment *μ* = 1.9×10^−16^ A⋅m^2^ for *T = *300 K in the first case, and *μ* = 2.1×10^−16^ A⋅m^2^ in the second case (Fig. 6B).

We next directly considered the distributions of observed orientations for each different field value. To eliminate any effect due to the possible misalignment of the camera with respect to the magnetic field, the distribution of orientations obtained for the cells at each magnetic field strength was first fitted with a variation of Eq. 1 that allowed for a constant offset in the orientation, which was then subtracted from all angle measurements. With the exception of the distributions obtained at *B = *0.0 mT and *B = *0.3 mT, the bias observed was always very small (less than 0.5°). Examples of orientation distributions obtained at 3 different field values after bias correction are shown in Fig. 6C. The shape of these distributions is consistent with the expected Boltzmann distribution, and fit of the data with either Eq. 6 (i.e. assuming 2D trajectories) or Eq. 4 (assuming 3D trajectories) returned a value for the quantity *µB*/*kT* for each magnetic field strength. At high field strengths the width of the orientation distributions approaches a finite limit of ∼5° likely representing the experimental resolution, thus the extracted values of *µB*/*kT* also reach a final limit. A fit to the linear regime of the data (B<2 mT) yields a magnetic moment of *μ* = 0.90×10^−16^ A⋅m^2^ assuming 2D trajectories or *μ* = 0.92×10^−16^ A⋅m^2^ assuming 3D trajectories (Fig. 6D).

#### Orientation correlations

**Figure 7:**
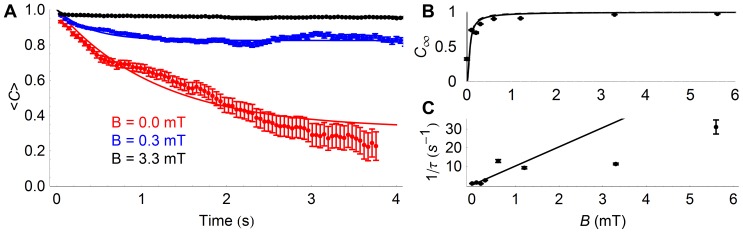
Orientation correlation function of cells placed in constant magnetic fields. A) Correlation of cell orientation over time for different values of the magnetic field, (mean ± standard error). Fit lines correspond to a modified worm like chain model (Eq. 15). B) Long time limit of the orientation correlation function. Fits correspond to the expectation of the paramagnetic model assuming either 2D trajectories (Eq. 17, continuous line), yielding a magnetic moment *μ* = 1.2×10^−16^ A⋅m^2^, or 3D trajectories (Eq. 16, continuous line), yielding *μ* = 1.5×10^−16^ A⋅m^2^. C) Persistence time of the orientation correlation function. The line represents a linear fit of the data for B<2 mT.

Orientation correlations calculated for the cell trajectories at different magnetic field strengths are shown in Fig. 7A. Although in the absence of an external magnetic field the correlation decays to a value close to zero, as soon as an external magnetic field is present, long-term correlation appears, as expected. The orientation correlation functions could be well fitted by assuming an exponential worm-like chain behavior at short time, and approach to a non-zero limit value at long time (Eq. 15). The long-time limit value of the orientation correlation, *C*(∞), increases with *B* as expected (Fig. 7B), and fit of the data according to Eq. 17 (i.e. assuming 2D trajectories) or Eq. 16 (i.e. assuming 3D trajectories) returns a value of the magnetic moment *μ* = 1.2×10^−16^ A⋅m^2^ in the first case and *μ* = 1.5×10^−16^ A⋅m^2^ in the second case. The short time decay rate of the orientation correlation, 1/*τ_P_*, also increases with *B* (Fig. 7C).

## Discussion

In this study we have performed magnetic moment measurements on a single *Magnetospirillum magneticum* AMB-1 population using six different methods, one based on the direct measurement of magnetosome size and the other five based on the analysis of cell trajectories as recorded with a phase contrast microscope. Because they are easy and inexpensive to implement, optical methods are generally attractive, and they are commonly used to measure the magnetic moment of magnetotactic cells. Our work allows a direct comparison between different optical methods, in order to determine which gives the most reliable and precise magnetic moment measurement.

The magnetic moment values measured by each method are summarized in table 1. They fall in the range 1 to 8×10^−16^ A⋅m^2^, which is higher than previously reported for this particular strain, with estimates that fell between 0.5 and 1×10^−16^ A⋅m^2^ [11], [18], [19]. In addition, while the average speed of the cells we measured, 49 µm/s, is exactly the same as what had been reported before, their average length, 3.21 µm, is slightly lower [17]. These discrepancies are not unexpected as growth conditions can considerably influence the characteristics of a cell population. In the case of magnetotactic bacteria in particular, composition of the growth medium and availability of oxygen have a considerable impact on the size and composition of the magnetic crystals, and therefore on the value of the cells’ magnetic moments [13], [17]. Thus the same strain may exhibit different morphological and magnetic characteristics from study to study. It is important to note that, for this reason, a meaningful comparison of the values of the magnetic moment as measured by different methods requires the same population of cells for all measurements, which is what has been done in this study.

**Table 1:** Average magnetic moment of *M. magneticum* AMB-1 cells measured by six different methods.

Method Type	Subtype	Name	Measured *μ* (A⋅m^2^)
Magnetosome Measurement			4.2±0.5×10^−16a^
Trajectory analysis	Relaxation	U-turn Analysis	4.3±0.4×10^−16a^
		Rotational Bias	7.1±0.2×10^−16b^
	Statistical	Trajectory Dispersion	1.9±0.2×10^−16c^
		Orientation Distribution	0.90±0.02×10^−16c^
		Orientation Correlation	1.2±0.3×10^−16c^

± s.e.m for measurements on multiple cells.^a^ Mean

^b^ Value and error returned by least-square fitting of data.

± standard deviation from alternate forms of the same general method (e.g. assuming either two-dimensional or three-dimensional trajectories).^c^ Value returned by least-square fitting of the data assuming two-dimensional trajectories

The trajectory analysis methods we used can be classified into two broad categories, termed “orientation relaxation” methods and “statistical” methods (table 1). The two orientation relaxation methods returned a magnetic moment *μ* = (5.7±2.0)×10^−16^ A⋅m^2^ (mean ± stdev) in good agreement with the value obtained by direct measurements of the magnetic crystals size on electron micrographs, *μ* = (4.2±0.5)×10^−16^ A⋅m^2^. Statistical methods, on the other hand, returned on average a 2- to 3- fold lower value, *μ* = (1.3±0.6)×10^−16^ A⋅m^2^ (mean ± stdev). Apart from the U-turn method, the same cell trajectories were used to implement all the trajectory-based analyses, thus this discrepancy cannot be explained by a difference in magnetic properties of the cells. Instead, the difference must result from either an incorrect assumption or a systematic error. In the case of the measurement of magnetosome dimensions from electron micrographs there is very little room for assumptions or systematic errors that may result in a 2 to 3-fold variation in the value of the magnetic moment. Imperfect alignment of the magnetic moments of individual crystals in the magnetosome might lead to a slight overestimate of the total magnetic moment of the cell, but probably by no more than ∼5% (i.e. the error if one crystal in the magnetosome has an orientation which is reversed from that of the others in the chain, something that was recently shown to occasionally happen for a different strain of magnetotactic bacteria [43]). In contrast, both the relaxation and the statistical methods have likely sources of systematic errors.

The two orientation relaxation methods used here are based on the measurement of the average relaxation time in the orientation of the cells in response to a perturbation. For the U-turn method, the perturbations are due to periodic reversals in the external magnetic field, while for the rotational bias the perturbations are due to small random thermal forces. In all cases, the relaxation time is governed by a balance between the restoring magnetic torque and a viscous torque, as expressed in Eq. 9, and therefore it is always proportional to the characteristic time 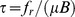. As a consequence, the precision on the measurement of the cell magnetic moment directly depends on how reliably one can estimate the rotational drag coefficient *f_r_*. Here we used a value of *f_r_* calculated by representing all cells as a cylinder of length *L = *3.21 µm. This represents an approximation for several reasons. First, the drag coefficient is expected to vary with the cube of cell length (Eqs. 10 and 11), and it is obvious from Fig. 2 that cell length has much variation within the population. Further *f_r_* of an AMB-1 cell might be greater than that of a cylinder with equal length: The spiral shaped body of the cells increases their surface area relative to a cylinder of the same length, and the presence of flagella at either end of the cell (each as long as the cell’s body [17]) may increase the total rotational friction applied on the cell [44]. Finally, the presence of a solid boundary near the cells, since all the trajectories analyzed here were recorded only a few microns above the coverslip, could influence the value of the rotational drag coefficient [41], [45], [46]. The exact influence of these effects is difficult to estimate precisely, yet they should be relatively minor, and neglecting them would be expected to produce an underestimate of the magnetic moment, and not an overestimate. Moreover, since orientation relaxation methods give average values of the magnetic moment that are in reasonable agreement with that obtained from the measurement of magnetosome dimensions (an agreement already noted in previous studies, performed on a different bacterial strain [22], [30]) it is likely that the cause of the discrepancy between the orientation relaxation methods and the statistical methods lies with the latter.

The term “statistical methods” refers here to methods relying on the assumption that the cell population behaves as a paramagnetic material, or that the distribution of cell orientation at equilibrium is correctly described by a Boltzmann distribution (Eq. 1). All consist in measuring the width of the distribution of orientations, either directly (orientation distribution method), or indirectly (trajectory dispersion and orientation correlation methods), and they all return the ratio *μB*/*kT*, i.e. the ratio of magnetic to thermal energy that regulates the degree of alignment of the cells with the external magnetic field. As shown in table 1, all three statistical methods delivered remarkably similar values of the magnetic moment, underlining their fundamental similarity. In the rest of the discussion, we concentrate on the orientation distribution method, for two reasons. First, it is the most robust of the three statistical methods: The trajectory dispersion method, which is most similar to statistical techniques described previously [21], [23], requires a decision about how the variance of *sinθ* is calculated, with different options leading to slightly different estimates of *μB*/*kT*; and the orientation correlation method, described for the first time in this study, requires the recording of many long trajectories in order to accurately calculate the long-time limit of the orientation correlation function. Second, and most importantly, the orientation distribution method gives the most direct way of testing the validity of the Boltzmann distribution captured in Eq. 1, which is the cornerstone of all statistical methods. In order to derive Eq. 1 several assumptions are necessary. Specifically, one needs to assume that thermal agitation is the only cause of deviation from alignment with the magnetic field; and that the system is at equilibrium. In addition, to derive actual angular distribution from Eq. 1, we made further assumptions, namely that all cells in the population had the same magnetic moment, and that their trajectories were either two- or three-dimensional, leading to Eqs. 6 and 4, respectively. We now examine these assumptions in more details.

**Figure 8:**
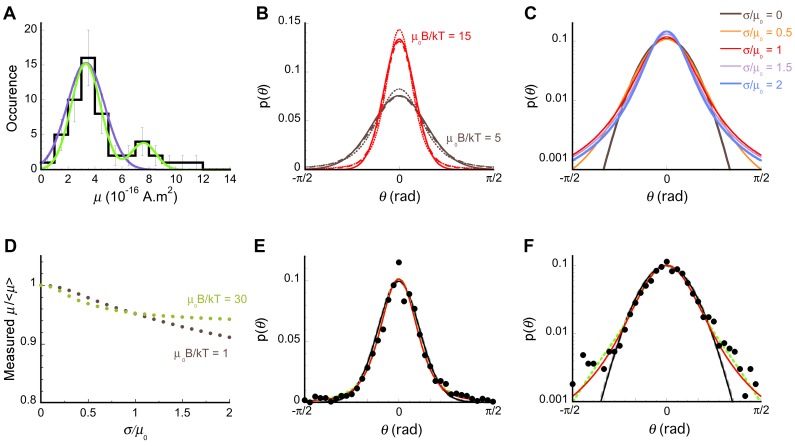
Distribution of magnetic moments. A) Experimental distribution of magnetic moment (black solid line), obtained by combining the values obtained by measurement of magnetosome size and those obtained using the U-turn method. Colored lines are fits of the data with either a single Gaussian distribution (purple line, *μ_0_* = 3.3×10^−16 ^A.m^2^, *σ* = 1.4×10^−16 ^A.m^2^) or the sum of two Gaussian distributions (green line, *μ_1_* = 3.3×10^−16 ^A.m^2^, *σ_1_* = 1.1×10^−16 ^A.m^2^, *μ_2_* = 7.6×10^−16 ^A.m^2^ and *σ_2_* = 0.9×10^−16 ^A.m^2^). B) Orientation distributions expected for cell populations with normal distributions of magnetic moment values, with *μ_0_B*/kT = 5 (brown lines) or *μ_0_B*/kT = 15 (red lines), and with *σ*/*μ_0_* = 0 (continuous lines), *σ*/*μ_0_* = 0.5 (dashed lines) or *σ*/*μ_0_* = 1 (dotted lines). C) Orientation distributions expected for a cell population with normally distributed magnetic moment values, *μ_0_B*/kT = 10 and different values of *σ*/*μ_0_*. D) Measured values of the magnetic moment, *μ*, obtained from fits of the orientation distributions assuming a monodisperse population of cells (i.e. using Eq. 6), normalized by the actual average value of the magnetic moment, <*μ*> (which is different from *μ_0_* for distributions with large standard deviation since the distribution does not extend to negative values) as a function of the actual normalized standard deviation of the magnetic moment distribution, *σ*/*μ_0_*. E) and F) Experimental orientation distribution measured at *B* = 0.3 mT (solid symbols). Lines indicate the best fit assuming 3D trajectories and a monodisperse population (Eq. 4, dashed grey line, *μ = *1.23×10^−16 ^A.m^2^) or 2D trajectories and either a monodisperse cell population (Eq. 6, solid black line, *μ = *1.16×10^−16 ^A.m^2^), a population made of cells with two different values of the magnetic moment (dashed green line, *μ_1_* = 0.43×10^−16 ^A.m^2^ and *μ_2_* = 1.90×10^−16 ^A.m^2^, <*μ*>* = *1.33×10^−16 ^A.m^2^), or a population with a normal distribution of magnetic moment values (Eq. 7, solid red line, returning *μ_0_* = 1.11×10^−16 ^A.m^2^ and *σ* = 0.97×10^−16 ^A.m^2^, corresponding to <*μ*> = 1.34×10^−16 ^A.m^2^).

Just as cells exhibit a length distribution (Fig. 2A), they exhibit a distribution of magnetic moments (Fig. 8A). Intuitively, a broad distribution of values for *μ* can be expected to result in a broadening of the cell orientation distribution, and therefore to an underestimate of the quantity *μB*/*kT* and in turn of the measured average magnetic moment. Indeed, a previous study that used statistical techniques to measure magnetic moment of cells stressed the importance of applying these methods only to individual cells for this reason [23]. The measured parameters used in statistical methods (*p*(*θ)*, *σ* ^2^_sin*θ*_ and *C*(∞)) have complex non-linear dependence on magnetic moment. For example, even in the relatively simple case of a normal distribution of magnetic moment, the distribution of orientations, which can be calculated by a convolution operation, cannot be reduced to a linear analytical form (Eq. 8). Thus the effect of a distribution of magnetic moment values on the cell orientation distribution is non-trivial and needs to be carefully examined.

The distribution of magnetic moments obtained by combining the results of the magnetosome measurement and U-turn methods (both well established non-statistical single cell methods, which individually returned very similar distributions) is shown in Fig. 8A. This distribution has a mean *<μ> = *4.3×10^−16 ^A.m^2^ and a standard deviation *σ = *2.4×10^−16 ^A.m^2^, and it is clearly right skewed (a feature visible also in a previous study of the same bacterial strain [18]). It can be adequately represented by a sum of two Gaussian distributions (Fig. 8A). We first consider the effect of a normal distribution of cellular magnetic moment, with mode *μ_0_* and standard deviation *σ.* Such a distribution of magnetic moments would result in a cell orientation distribution described by Eq. 8. When comparing this distribution to that of a monodisperse cell population (described by Eq. 6), we see that it is narrower at small angles, but broader at large angles (Fig. 8B,C). For values of *σ* less or on the order of *μ_0_*, these modifications are modest, and insignificant compared to those resulting from a 3-fold increase in *μ_0_* (Fig. 8B). Attempts to fit the complex distributions expected for normally distributed magnetic moments with the monodiperse equation (Eq. 6) lead to an underestimate of the average magnetic moment, <*μ>*, as expected (Fig. 8D). However, for *σ/μ<2* this discrepancy is always less than 10% (Fig. 8D). Considering the possibility that the cells exhibit a range of magnetic moments therefore does not significantly changes the estimate of the average magnetic moment obtained using a cruder analysis. Nevertheless, it does lead to a better fit of the experimental distribution, where the tails of the distribution are accounted for by a standard deviation on the order of *σ* ∼ *μ_0_* (Fig. 8E,F). The orientation distribution is also well fitted by assuming the more realistic case (given the actual experimental distribution of magnetic moments, Fig. 8A) of two monodisperse populations, but again the average magnetic moment is very similar to that obtained using the simple monodisperse model (Fig. 8E, F).

Another assumption that needs to be examined is the assumption that the cells undergo two-dimensional motion. We therefore also analyzed our data under the assumption that the 2D trajectories we observed were only projections in the focal plane of 3D trajectories (Figs. 5B, 5D and 6B). In particular, Fig. 8E,F shows a comparison between the fit of the magnetic moment orientation distribution assuming 2D (solid black line) or 3D (dashed grey line) trajectories. These fits are almost indistinguishable, and in all cases we found that value of *μ* that is obtained by assuming 3D trajectories is higher than the value obtained assuming 2D trajectories, but only slightly (∼ 10%). Thus the estimate of the magnetic moment depends only weakly on the trajectory dimensionality. Because of the preferred location of the cells close to the glass coverslip, the extended lengths of their trajectories and the fact that their projecte length is always within 15% of their maximum length, it is in fact likely that the trajectory recorded were indeed true two-dimensional trajectories, and for this reason the values of *μ* listed in Table 1 are those obtained assuming 2D trajectories.

In conclusion, the estimate of the average value of the magnetic moment of a cell population using our cell trajectory analysis methods is robust against different assumptions that can be made about the width of the cell magnetic moment distribution and dimension of their trajectories. In consequence, the larger than expected distribution of cell orientations that we observed assuming a monodisperse population of cells undergoing 2D motion can be explained neither by a distribution of magnetic moments nor by three-dimensional trajectories.

It had previously been reported that magnetotactic bacteria displayed larger than expected departure from magnetic alignment considering the presumed value of their magnetic moment [23], [27]. Our study reinforces this finding since our experiments were done with a single cell population. Since our observations cannot be explained by a distribution of magnetic moment values, they are consistent with the presence of non-thermal energy sources not accounted for in the paramagnetic model, which affect cell orientation and raise the effective temperature of the system, thus lowering the value of the ratio *μB*/*kT*. Non-thermal stochastic forces have been shown to be important in living systems, which are not in thermodynamic equilibrium, and where non-thermal fluctuations can be up to two orders of magnitude larger than thermal fluctuations [27], [47]–[50]. For example, the severity of random fluctuations observed in intracellular diffusion [48], cell membrane movement [51], and vibration of chromosomal loci [52], is increased in the presence of ATP hydrolysis. In the case of magnetotactic bacteria, it has been remarked that flagellar movements could affect orientation, adding an additional source of energy that would not be accounted for by the paramagnetic model [4], [23], [27]. This effect could be compounded by collision with other bacteria (alive or dead) and with interactions through hydrodynamic forces to add an additional level of non-thermal stochastic noise. This proposition is supported by the fact that a solution containing live cells exhibits a larger orientation distribution than a solution of dead cells [27]. In that case an effective temperature 10–20% larger than the actual temperature was necessary to explain observations [27]. The effect we observe here is stronger, where an effective temperature of *T ∼*750 K would be necessary to explain the value of the ratio *μB*/*kT* we observed.
